# Pseudo loss of resistance in epidural space localization: A complication of subcutaneous emphysema or simply a faulty technique

**DOI:** 10.4103/1658-354X.76464

**Published:** 2011

**Authors:** Amit Jain

**Affiliations:** *Department of Anaesthesia & Intensive Care, Post Graduate Institute of Medical Education and Research, Chandigarh, India*

Sir,

We read with interest the Letter to the Editor, “Pseudo loss of resistance in epidural space localization”.[1] The inference of the authors seems to be simple, but a closer look reveals the omission of many implicating factors that would have contributed to the false loss of resistance (LOR). Therefore, we dispute the conclusions. It appears that the needle was merely inserted into the supraspinous (SS) or interspinous (IS) ligament before the LOR with glass syringe was attempted. Therefore, we strongly believe that the pseudo LOR perceived by the authors could be primarily due to the needle entering the paraspinous muscles with subcutaneous emphysema forming air pockets in the deeper planes may be a secondary factor. We believe that the formation of air pockets within the tough ligamentum flavum (LF) and IS ligaments following subcutaneous emphysema is impossible, though cystic degeneration of these ligaments is well described in the literature.[1,2]

A LOR sensation may be perceived, even though the epidural needle tip is not located in the epidural space. If needle is merely inserted into the SS ligament and then LOR or hanging–drop insertion is begun, an increased chance of false release seems likely. In particular, this may occur if the needle enters the IS ligament at an oblique angle. The needle tip will exit the ligament into the soft tissue on the opposite side with a LOR feel. As the authors perceived false LOR at the depth of 3.5cm twice, while epidural space was localized at 4.5 cm only on third attempt, the above-mentioned explanation seems to be more appropriate. Furthermore, the possibility of midline air pockets in any of the three ligaments traversed by the epidural needle could not be explained anatomically. Multiple causes of collections of air in the spinal canal including epidural abscess, iatrogenic interventions, asthma attacks, violent vomiting or coughing, blunt chest trauma, physical exertion, and chest tube replacement have beendescribed.[3,4] Epidural emphysema associated with pneumothorax and pneumomediastinum has also been reported.[4] The absence of a fascial barrier between the posterior mediastinum and neck allows the air to migrate from these spaces into the epidural space through the intervertebral foramina.[3] However, in medline research, we did not find any reports of the formation of pneumatic cysts in the any of the spinal ligaments following such an event. The CT technique, which successfully demonstrated the epidural pneumatosis,[3] could have been valuable in diagnosing such midline air pockets, should they occur.

Clinically, one method to differentiate between soft tissue and the epidural space is to inject saline with a small air bubble in the syringe. If the saline is being injected into the epidural space the bubble will not compress. In contrast, the bubble will compress if the saline is being injected into soft tissue.[5] However, certainly the presence of air pockets in the paraspinous muscles may results in bubble compression sign non-appreciable.

In the described scenario, a few anatomical and technical considerations are important for the successful localization of the epidural space by midline approach.[5]

1. The LF lies immediately posterior to the epidural space.
2. Once the epidural needle is engaged in the IS ligament the epidural needle will stay firmly at midline without support, indicating correct placement into the IS ligament.
3. The LF is tougher than the IS ligament. Thus, as the needle is advanced further and is in midline, the tissues feel firmer upon needle tip entry into the LF. Sometimes crepitation is felt or even heard as the LF is encountered. Placing the needle (with stylet) into the LF before attaching the syringe or placing solution into the needle hub allows an improved appreciation of epidural anatomy for the operator.
4. Although, the chosen technique is largely a matter of training and personal experience, inserting the needle to the LF and then attaching a 3-to-5ml glass syringe filled with 2 ml of saline and a small (0.25 ml) air bubble seems to be the preferred method of carrying out the LOR technique.[6]
